# Targeted high mean arterial pressure aggravates cerebral hemodynamics after extracorporeal resuscitation in swine

**DOI:** 10.1186/s13054-021-03783-3

**Published:** 2021-11-14

**Authors:** Yael Levy, Alice Hutin, Fanny Lidouren, Nicolas Polge, Rocio Fernandez, Matthias Kohlhauer, Pierre-Louis Leger, Guillaume Debaty, Keith Lurie, Lionel Lamhaut, Bijan Ghaleh, Renaud Tissier

**Affiliations:** 1 INSERM, IMRB, Univ Paris Est Créteil, 94010 Créteil, France; 2 IMRB, AfterROSC Network, Ecole Nationale Vétérinaire d’Alfort, 7 Avenue du Général de Gaulle, 94700 Maisons-Alfort, France; 3 Assistance Publique-Hôpitaux de Paris, SAMU de Paris-ICU, Necker University Hospital, Université de Paris, 75015 Paris, France; 4 Department of Small Animal Medicine and Surgery, Faculty of Veterinary Medicine, Catholic University of Valencia, 46001 Valencia, Spain; 5 Assistance Publique-Hôpitaux de Paris, Hôpital Trousseau, Université de Paris, Sorbonne Université, 75012 Paris, France; 6 Department of Emergency Medicine, CNRS, TIMC Laboratory - UMR 5525, Grenoble Alpes University Hospital, University Grenoble Alpes, Grenoble, France; 7 Hennepin Healthcare Research Institute, Minneapolis, MN USA

**Keywords:** Cardiac arrest, Resuscitation, Extracorporeal circulation, Epinephrine, Blood pressure

## Abstract

**Background:**

Extracorporeal cardiopulmonary resuscitation (E-CPR) is used for the treatment of refractory cardiac arrest. However, the optimal target to reach for mean arterial pressure (MAP) remains to be determined. We hypothesized that MAP levels critically modify cerebral hemodynamics during E-CPR and tested two distinct targets (65–75 vs 80–90 mmHg) in a porcine model.

**Methods:**

Pigs were submitted to 15 min of untreated ventricular fibrillation followed by 30 min of E-CPR. Defibrillations were then delivered until return of spontaneous circulation (ROSC). Extracorporeal circulation was initially set to an average flow of 40 ml/kg/min. The dose of epinephrine was set to reach a standard or a high MAP target level (65–75 vs 80–90 mmHg, respectively). Animals were followed during 120-min after ROSC.

**Results:**

Six animals were included in both groups. During E-CPR, high MAP improved carotid blood flow as compared to standard MAP. After ROSC, this was conversely decreased in high versus standard MAP, while intra-cranial pressure was superior. The pressure reactivity index (PRx), which is the correlation coefficient between arterial blood pressure and intracranial pressure, also demonstrated inverted patterns of alteration according to MAP levels during E-CPR and after ROSC. In standard-MAP, PRx was transiently positive during E-CPR before returning to negative values after ROSC, demonstrating a reversible alteration of cerebral autoregulation during E-CPR. In high-MAP, PRx was negative during E-CPR but became sustainably positive after ROSC, demonstrating a prolonged alteration in cerebral autoregulation after ROSC. It was associated with a significant decrease in cerebral oxygen consumption in high- versus standard-MAP after ROSC.

**Conclusions:**

During early E-CPR, MAP target above 80 mmHg is associated with higher carotid blood flow and improved cerebral autoregulation. This pattern is inverted after ROSC with a better hemodynamic status with standard versus high-MAP.

**Supplementary Information:**

The online version contains supplementary material available at 10.1186/s13054-021-03783-3.

## Introduction

Out-of-hospital cardiac arrest is the leading cause of mortality in western countries, with an extremely low survival and poor neurological outcome [1]. In order to improve the resuscitation rate, extracorporeal cardiopulmonary resuscitation (E-CPR) is currently suggested, when conventional cardiopulmonary resuscitation (CPR) fails to achieve return of spontaneous circulation (ROSC). It allows restoring organ perfusion whilst the underlying etiology is identified and properly treated [2, 3]. In the clinical arena, E-CPR has been shown to improve survival to hospital discharge and functional status when implemented early, as compared with standard prolonged resuscitation [4, 5]. Nevertheless, the ideal hemodynamic management remains unclear during E-CPR, e.g., regarding optimal mean arterial pressure (MAP) target.

After conventional CPR, several retrospective studies have reported a correlation between favorable outcome and higher MAP target after ROSC [6–8]. Cerebral blood flow and cerebral autoregulation are indeed known to be impaired during the first 72 h following cardiac arrest in resuscitated patients [9–11]. International guidelines recommend a MAP > 65 mmHg in all resuscitated patients [12, 13]. However, there is no few specific data regarding cerebral hemodynamics at different MAP levels during E-CPR to our knowledge, despite its relevance as a determinant of cerebral outcome. In swine, no difference on systemic hemodynamics and microcirculation parameters was observed with a MAP set at 65–70 versus 80–85 mmHg but cerebral hemodynamics and autoregulation were not evaluated [14]. A specific investigation of these parameters is of importance since brain circulation is peculiar with a proper autoregulation and cerebral dramatic metabolic changes after cardiac arrest.

Accordingly, the goal of the present study was to compare the effects of two different MAP targets on cerebral hemodynamics and metabolism during E-CPR (70 ± 5 vs. 85 ± 5 mmHg). We used a swine model of cardiac arrest with a prolonged no-flow period of 15 min, in order to mimic a severe hypoxic-ischemic injury that could be observed after refractory cardiac arrest in patients submitted to E-CPR. We started E-CPR directly after the end of the no-flow period to assess its proper effect without other confounding factors secondary to conventional CPR or chest compression.

## Methods

All experiments were reviewed and approved by the ethical committee ComEth Anses-EnvA-UPEC (Committee No. 16, Project #23076-2019112616472793). All procedures were conducted in accordance with the European Community Standards on the Care and Use of Laboratory Animals.

### Animal preparation

Twelve female swine (27–35 kg) were anesthetized with a mixture of zolazepam and tiletamine (10 mg/kg, i.m.) followed by propofol (1 mg/kg i.v.). After endotracheal intubation, animals were submitted to conventional mechanical ventilation (tidal volume = 9 ml/kg; FiO2 = 30%; respiratory rate = 20 breaths/min; positive end-expiratory pressure = 5 cmH_2_O). Ventilation parameters were modified when needed to maintain normocapnia et normoxia. Anesthesia was maintained during the instrumentation phase by a continuous administration of propofol (10 mg/kg/h). Animals also received methadone (0.3 mg/kg i.m.) and rocuronium (1 mg/kg i.v.) for analgesia and muscular paralysis, respectively.

Two catheters (9 Fr) were introduced by the Seldinger technique through the right femoral vein and artery for the continuous monitoring of right atrial and systemic arterial blood pressure, respectively. The same technique was used to insert a catheter into the jugular vein for the evaluation of jugular venous oxygen saturation (SjvO2). A 3 mm blood flow probe (PS-Series Probes®, Transonic, NY, USA) was placed around the internal carotid artery to monitor carotid blood flow (CBF). A pressure gauge (Millar®, SPR-524, Houston, TX, USA) was inserted into the cerebral cortex after craniotomy to monitor intracranial pressure (ICP). Cerebral oxygen saturation was continuously monitored by near infrared spectroscopy (NIRS; INVOS™ 5100C Cerebral/Somatic Oximeter, Medtronic®). Two guidewires were also placed into the left femoral artery and vein for the further insertion of cannulas for extracorporeal membrane oxygenation (ECMO) after induction of cardiac arrest. Unfractionated heparin (100 UI/kg i.v. bolus) was administered immediately after instrumentation. In order to compensate fluid loss, an i.v. infusion of fluid (Ringer lactate, 10 ml/kg) was performed during instrumentation.

### Cardiac arrest and E-CPR protocol

After a period of stabilization, ventricular fibrillation (VF) was induced by a pacemaker catheter introduced into the right ventricle through the venous femoral sheath (A/C 10 V). VF was left untreated during 15 min of no-flow, with no mechanical ventilation. During this period, two 21 and 15 Fr cannulas were mounted around the guidewires previously inserted into femoral vein and artery, respectively (HLS Cannulaes ®, Maquet, Rastatt, Germany). At the end of the 15 min of untreated VF, E-CPR was started with a pump flow of 40 ml/kg/min. The extracorporeal life support circuit included a console, a centrifugal pump (Deltastream® DP3 Pump Heads (Medos Medizintechnik AG, Stolberg,Germany), a membrane oxygenator (PLS-i Oxygenator®, Maquet, Rastatt, Germany) and a tubing set (PLS Set®, Maquet, Rastatt, Germany). The membrane oxygenator was connected to a mechanical gas blender system (Sechrist Model 20,090®, Sechrist, Anaheim, Calif). The gas flow was adjusted to target a CO_2_ blood partial pressure between 35 and 45 mmHg. After 30 min of E-CPR, defibrillation attempts were started (150 J). Mechanical ventilation was resumed immediately after ROSC with the initial parameters. Fluid administration was standardized in all animals with an administration of 15 and 30 ml/kg of Ringer Lactate immediately after E-CPR initiation and over the two hours following ROSC, respectively. All animals were followed during two hours after ROSC. Then, they were euthanized.

### Experimental groups

**Fig. 1:**
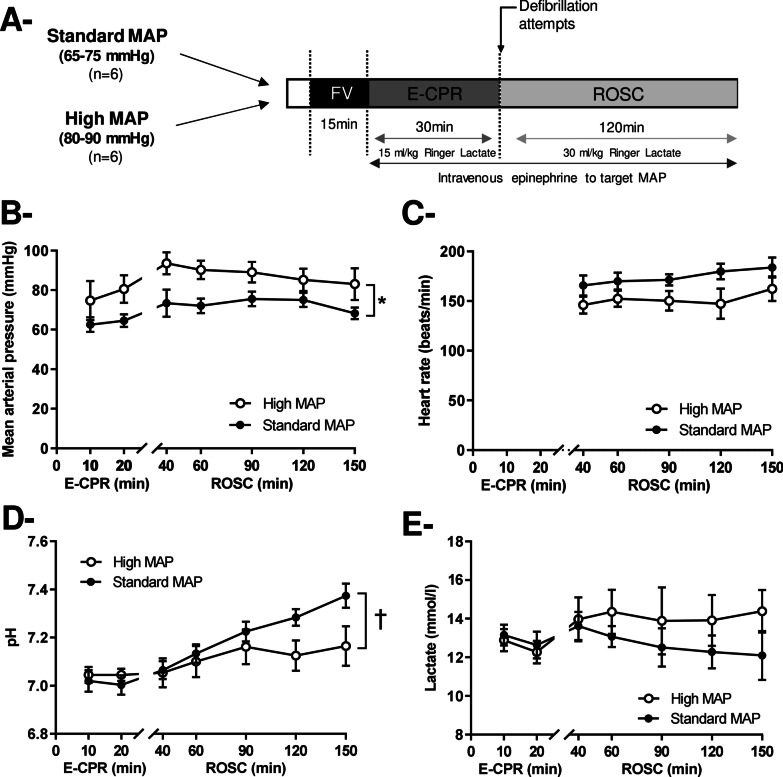
Experimental protocol (**Panel A**), mean arterial pressure (MAP,** Panel B**), heart rate (**Panel C**) and blood pH (**Panel D**) and lactate levels (**Panel E**) after cardiac arrest. *MAP* mean arterial pressure, *E-CPR* extra-corporeal cardiopulmonary resuscitation, *ROSC* return of spontaneous circulation; *p < 0.05 for the group effect of the two-way analysis of variance throughout follow-up; ^†^*p* < 0.05 for the group x time interaction effect of the two-way analysis of variance; *p* values of the contingency table of the two-way analysis are presented in Additional file 1: Table S1

As illustrated by Fig. 1a, animals were randomly divided in two experimental groups submitted to different MAP targets after cardiac arrest, *i.e.*, either 65–75 (standard MAP) or 80–90 mmHg (high MAP). This target was achieved by epinephrine administration at adjusted regimen of administration before and after ROSC. We used epinephrine as it is usually recommended during CPR (before ROSC). After cardiac arrest, we targeted a temperature of 37.0 ± 0.5 °C using thermal pads and infra-red light.

### Investigated parameters

Heart rate (HR), systolic and diastolic blood pressure, MAP and right atrial pressure were continuously monitored and recorded. CBF, ICP and cerebral NIRS were also monitored and recorded throughout the protocol. Cerebral perfusion pressure was calculated as the difference between MAP and ICP. We also calculated the pressure reactivity index (PRx) as the correlation coefficient between ICP and MAP. It was calculated at baseline and 20, 60, 90, 120 and 150 min after E-CPR institution during 40 consecutive 6-secondes averages of ICP and MAP, as previously reported [15]. A positive PRx value reflects a nonreactive vascular bed whereas a negative or zero value reflects a normally reactive vascular bed [15, 16].

Arterial and venous blood pH, gases (O_2_ and CO_2_ partial pressure [pO_2_ and PCO_2_, respectively]), lactates and hemoglobin levels were measured at baseline, during E-CPR (*t* = 10 and 20 min) and after ROSC (*t* = 40, 60, 90, 120 and 150 min). The oxygen consumption of the brain hemisphere was approximated using the following equation: VO_2_ (ml O_2_/min/kg) = (CaO_2_–CvO_2_) × CBF, where CaO_2_ is arterial oxygen content, CvO_2_ is internal jugular venous oxygen content, and CBF is carotid blood flow. CvO_2_ was calculated as follows: CvO_2_ (ml/l) = [(1.34 × Hb(g/dl) × SvjO_2_) + (0.0031 × PvjO_2_)] × 10, where SvjO_2_ is internal jugular venous oxygen saturation and PvjO2 is internal jugular venous oxygen tension.

Blood levels of creatinin, alanine aminotransferases (ALT), troponin I and PS100 were also evaluated at baseline and 150 min after cardiac arrest, as well as interleukin-1α (IL-1α) and interleukin-1β (IL-1β; ELISA kits, R&D Systems, Minneapolis, MN).

### Statistical analysis

Data were expressed as mean ± SEM. Data normal distribution was verified by a Shapiro–Wilk normality test. At baseline, values were compared among groups using a Student *t*-test. After cardiac arrest, all parameters with repeated measures were compared among groups using a two-way analysis of variance for repeated measures (timing = 10, 20, 40, 60, 90, 120 and 150 after the onset of E-CPR). The p values of the corresponding time, group and time x group interaction effects are shown in Additional file 1: Table S1. We only considered the global effects of the ANOVA and did not perform post-hoc comparisons at each time point, in order to avoid multiple comparisons. The parameters with only one measurement at 150 min after cardiac arrest were compared among groups with a Student *t*-test. A value of *p* < 0.05 was considered statistically significant. All statistical analyses were performed using GraphPad Prism® software (GraphPad® Software, La Jolla, CA, USA).

## Results

Twelve swine were enrolled in the present study, *i.e.*, 6 in both standard- and high-MAP groups. Body weights and temperature were not different among groups (30 ± 2 vs 28 ± 1 kg and 37.9 ± 0.3 vs 37.5 ± 0.3 °C, respectively), as well as hemodynamic and biochemical parameters at baseline (Table 1). After 30 min of E-CPR, all animals achieved ROSC after the first electric shock, except one animals which required a second shock in the standard-MAP group.

**Table 1:** Baseline values of investigated parameters

Baseline parameters	Standard-MAP N = 6	High-MAP N = 6	*p*
Systemic hemodynamic			
Heart rate (bpm)	78 ± 5	82 ± 9	0.55
MAP (mmHg)	95 ± 16	98 ± 14	0.54
Cerebral hemodynamic			
Intracranial pressure (cm H_2_O)	10 ± 1	10 ± 1	0.84
Cerebral perfusion pressure (mmHg)	84 ± 7	87 ± 6	0.73
Carotid blood flow (ml/min)	156 ± 27	137 ± 8	0.52
Cerebral oxygen consumption (ml O_2_/min/kg)	45 ± 3	48 ± 7	0.63
NIRS cerebral oxygen saturation (%)	62 ± 5	56 ± 2	0.68
Blood biochemistry			
pH	7.46 ± 0.02	7.42 ± 0.01	0.21
PaO_2_ (mmHg)	158 ± 3	152 ± 7	0.68
PaCO_2_ (mmHg)	37 ± 2	40 ± 2	0.22
HCO_3_^−^ (mmol/L)	25 ± 1	25 ± 2	0.88
Lactate (mmol/L)	2.5 ± 0.5	4.4 ± 1.4	0.34
Hemoglobin (g/dL)	9.2 ± 0.4	9.6 ± 0.4	0.52

Data are presented as Mean ± SEM

*MAP* Mean arterial pressure, *NIRS* near infrared spectroscopy, *PaO*_*2*_ arterial oxygen partial pressure, *PaCO*_*2*_ arterial carbon dioxide partial pressure

### Systemic hemodynamic and blood gases

As illustrated by Fig. 1b, MAP was maintained at the expected levels in both standard-MAP (65–75 mmHg) and high-MAP (80–90 mmHg). Heart rate was not significantly different among groups (Fig. 1c; Additional file 1: Table S1). Right atrial pressure was not different in standard-MAP vs high –MAP during E-CPR (e.g., 16 ± 2 vs 16 ± 3 mmHg at t = 20 min after cardiac arrest) or after ROSC (11 ± 1 vs 10 ± 4 mmHg at *t* = 150 min after cardiac arrest). The total amount of epinephrine administered to achieve MAP target during the first 30 min of E-CPR was lower in standard-MAP vs high –MAP group (51 ± 13 vs 102 ± 21 µg/kg over 30 min). It was also the case after ROSC, until the end of the protocol (23 ± 9 vs 145 ± 92 µg/kg over 120 min in standard-MAP vs high-MAP group).

A strong metabolic acidosis was observed in both groups after cardiac arrest, as evidenced by low blood pH (Fig. 1d), high blood lactate levels (Fig. 1e) and decreased bicarbonate blood levels (e.g., 16 ± 1 and 12 ± 1 mmol/L in standard-MAP and 17 ± 1 and 11 ± 1 mmol/L in high-MAP at *t* = 10 and 150 min after cardiac arrest). After ROSC, blood pH was significantly higher in standard- versus high-MAP. Lactate blood levels tended to decrease in standard- versus high-MAP but this did not reach statistical significance. PaO_2_ and PaCO_2_ did not significantly differ between groups throughout the protocol (data not shown).

### Cerebral hemodynamic

**Fig. 2:**
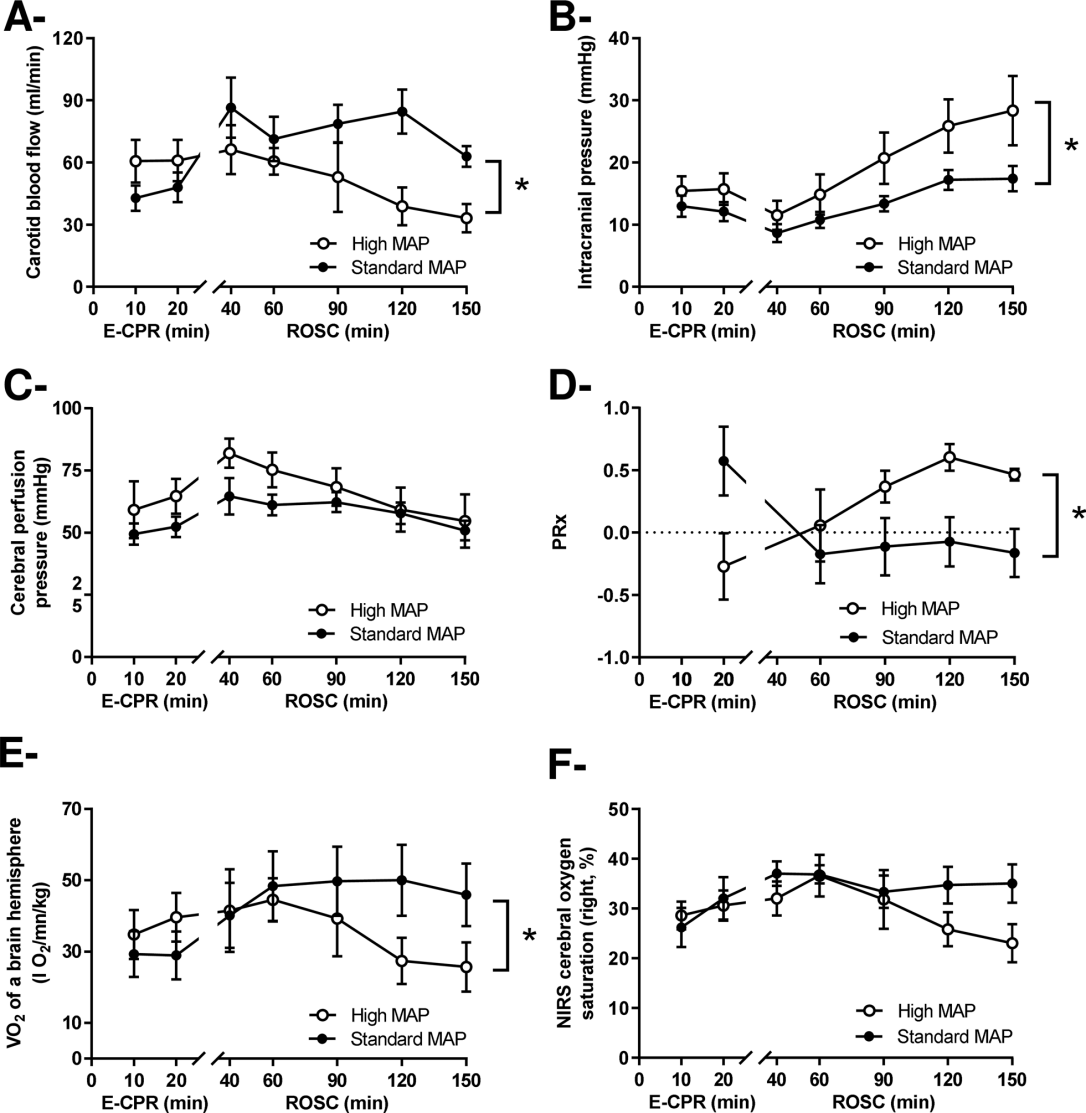
Cerebral hemodynamic parameters after cardiac arrest, including carotid blood flow (**Panel A**), intracranial pressure (**Panel B**), cerebral perfusion pressure (**Panel C**), pressure reactivity index (PRx,** Panel D**), Oxygen consumption of a brain hemisphere (**Panel E**) and Cerebral oxygen saturation assessed by near-infrared spectroscopy (NIRS;** Panel F**). *MAP* mean arterial pressure; *NIRS* near infrared spectroscopy, *PRx* pressure reactivity index (correlation coefficient between intracranial pressure and MAP); *ROSC* return of spontaneous circulation, *VO*_*2*_ oxygen consumption; *p < 0.05 for the group effect of the two-way analysis of variance throughout follow-up; ^†^*p* < 0.05 for the group x time interaction effect of the two-way analysis of variance; *p* values of the contingency table of the two-way analysis are presented in Additional file 1: Table S1

During early E-CPR, CBF was greater in high vs standard-MAP (i.e., before ROSC; Fig. 2a). After ROSC, CBF was conversely lower in high- versus standard-MAP (*p* < 0.05 for time x group interaction, Additional file 1: Table S1). This was associated with greater amplitude in ICP increase with high- vs standard-MAP after ROSC (*p* < 0.05 for time x group interaction (Fig. 2b). This led to similar cerebral perfusion pressure among groups at the end of the follow-up Fig. 2c), despite MAP differences.

As illustrated in Fig. 2d, the PRx autoregulation index became transiently positive with standard-MAP just after the onset of E-CPR (*t* = 20 min), indicating altered cerebral autoregulation. Conversely, PRx was negative during early E-CPR with high-MAP but became sustainably positive after ROSC. If we consider a threshold of + 0.2 as a marker of dramatic autoregulation disruption, 5/6 versus 1/6 animals demonstrated values above that threshold in standard vs high-MAP during E-CPR, respectively. At the end of the follow-up (*t* = 150 min), those numbers were conversely 1/6 and 6/6 in standard vs high-MAP, respectively. This was consistent with the significant decrease in cerebral oxygen consumption in high- versus standard-MAP after ROSC (Fig. 2e). In the same line, cerebral oxygen saturation assessed by NIRS tended to be improved after ROSC with standard versus high-MAP group (Fig. 2f), even if statistical significance was not achieved.

### Markers of multi-organ failure and inflammation

**Fig. 3:**
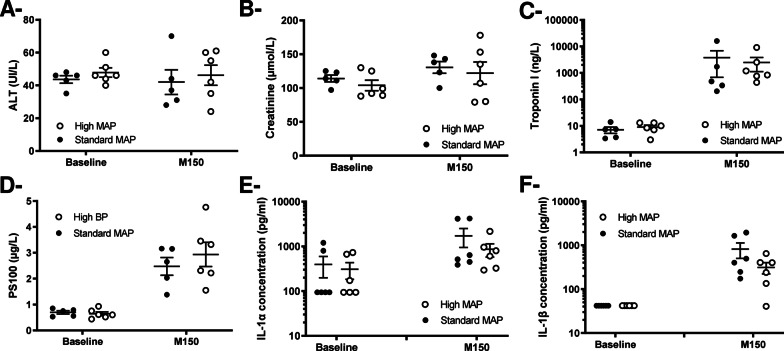
Blood levels of biochemical markers of multi-organ failure (and inflammation after cardiac arrest (i.e., alanine aminotransferases [ALT,** Panel A**], creatinine [**Panel B**], troponin I [**Panel C**], PS100 [**Panel D**], interleukin [IL]-1α [**Panel E**] and IL-1β [**Panel F**]). *MAP* mean arterial pressure; *M150* time = 150 min after the onset of E-CPR

As illustrated in Fig. 3a–d, blood levels of ALT, creatinine, troponin and PS100 similarly increased in both groups after cardiac arrest, demonstrating liver, renal, cardiac and neurological injuries, respectively. The blood levels of IL-1α and IL-1β also similarly increased after cardiac arrest in both groups (Fig. 3e–f).

## Discussion

In our experimental model of E-CPR, high-MAP transiently improved cerebral hemodynamics after cardiac arrest, as compared to standard-MAP. It was evidenced by higher CBF and lower PRx during early E-CPR. However, this beneficial effect was lost after ROSC and high MAP became deleterious with a dramatic deterioration of cerebral hemodynamics, including lower CBF, positive PRx, increased ICP and deteriorated cerebral oxygen consumption and saturation vs standard-MAP. High-MAP also worsened metabolic acidosis as compared to standard-MAP, despite similar blood levels of biomarkers of end-organ of dysfunction at the end of the follow-up.

In the literature, it is well admitted that cerebral perfusion and autoregulation are strongly impaired and associated with unfavorable neurological outcome after cardiac arrest [9, 11, 17]. Cerebral blood flow is then often believed to directly depends upon perfusion pressure, supporting the putative interest of high-MAP targets. However, MAP support requires the administration of catecholamines exerting their own effects on microvascular beds and cerebral blood flow. It is particularly true with epinephrine which was shown to decrease cerebral microvascular blood flow and increase the severity of cerebral ischemia in swine submitted to cardiac arrest and conventional CPR [18]. This could explain why high-MAP was beneficial during the very early phase of E-CPR but became clearly detrimental after ROSC in our study. The decrease in cerebral oxygen consumption and CBF could then be related to altered microcirculation and ICP increase, with increased cerebral vascular resistance and cerebral swelling.

In the present study, PRx analyses also demonstrate altered cerebral autoregulation with high-MAP after ROSC, with a positive correlation between ICP and MAP after ROSC. Interestingly, PRx values above + 0.2 were shown to predict poor neurological outcome with a strong specificity and sensitivity in patients after conventional CPR [19]. When compared to standard-MAP, the high-MAP strategy was therefore clearly deleterious after ROSC, although transiently improving cerebral hemodynamic during the early phase preceding ROSC in our study. One could argue that a double approach should be proposed with a high-MAP target during early E-CPR and then a standard-MAP after ROSC. Unfortunately, such a condition was not included in our randomization plan but clearly deserves further investigation. An investigation with norepinephrine rather than epinephrine would also be relevant after ROSC. Beyond the vasotensive effect of those drugs, the worsening of cerebral hemodynamics with high-MAP could also be indirectly related to altered venous drainage and increased venous congestion and vascular leakage during E-CPR. However, this should not be the major mechanism since ECMO flows, fluid administration and right atrial pressure were similar in standard- and high-MAP groups.

Importantly, the current recommendations for hemodynamic managements of cardiac arrest patients were mostly raised after conventional CPR (i.e., not specifically following E-CPR). A MAP level above 65 mmHg with prevention of hypotension is then proposed [12]. Hypotension is indeed clearly associated with poor neurological outcome [6, 7, 20], while observational studies showed an improved neurological outcomes with MAP levels set at 70–75 mmHg [7, 8]. Importantly, randomized studies did not demonstrate a deleterious effect of higher MAP in those conditions. For instance, the neurological injury evaluated by neural specific enolase blood levels was not different with a MAP of level of 65–75 versus 80–100 mmHg after ROSC [21]. In another study, similar extents of anoxic brain damage were observed with a MAP target of 85–100 mmHg, with a mixed venous O_2_ saturation of 65–75%, when compared to a 65 mmHg MAP strategy [22]. All these studies were obtained after conventional CPR and one would speculate different situations in patients with E-CPR, due to the increased severity of the anoxic brain injury, as suggested by our present findings. This difference should not be so critical for other organ injury and systemic hemodynamics as we did not observe much difference on systemic hemodynamics and other biomarkers, except a significantly higher blood pH with standard- versus high-MAP after ROSC. This is consistent with the results obtained by Fritz et al. [14] in swine submitted to prolonged cardiac arrest and E-CPR with two different MAP targets (65–70 mmHg vs 80–85 mmHg) during 6 h after ECMO initiation. The latter study did not assess cerebral hemodynamics to our knowledge. Longer follow-up duration would also evidence differences on the longer term.

In summary, our experimental study supports the use of a 65–75 mmHg MAP target in patients under ECMO, at least after ROSC. Specific clinical trials are still required but our findings, along with most clinical data in patients resuscitated after convention CPR, indeed demonstrates the lack of benefit, or even the deleterious effect, of high- vs standard-MAP target. We should point that this putative recommendation could only be relevant for MAP target with vasopressors, which does not mean that similar levels of MAP could lead to similar observations in patients weaned from vasotensive agents.

### Study limitations

Our present study presents several limitations. As already discussed, we used epinephrine to achieve the MAP target in both groups since we initially focused on the early E-CPR preceding ROSC, for which epinephrine is likely the most relevant drug. It would have been difficult to interpret the results if using different drugs after ROSC, e.g., dobutamine and norepinephrine. If norepinephrine is usually recommended after ROSC [12], a recent Cochrane review did not find any difference in mortality with six different vasopressors assessed in 3497 patients presenting hypotensive shock [23]. Second, another important limitation is the short follow-up duration of the study, which did not allow the evaluation of the long term neurological outcome. Cerebral imaging or histological analyses would also have been of high interest but they were not conducted in the present study. We can only raise our conclusions on surrogate markers such as the PRx as an autoregulation parameter. Third, we only follow animals during a short period that makes the analysis of biomarkers data difficult. Finally, such experimental studies are typically done using a limited number of animals. Since we only investigated females, it is also uncertain that the results could be generalized to males.

## Conclusions

In an experimental swine model of refractory cardiac arrest treated by E-CPR, a high-MAP target improved hemodynamics during the early phase of E-CPR preceding ROSC, when compared to standard-MAP. Conversely, high-MAP became highly deleterious after resuscitation. This could support a combined strategy”, with an initial aggressive MAP control followed by a more conservative strategy after E-CPR.

## Supplementary Information

**Additional file 1**. Supplemental Table I: P values of the contingency table of the two-way analysis of variance (ANOVA) for repeated measures with group, time effect and group x time interaction effects for the different investigated parameters after cardiac arrest.

## Data Availability

The datasets used and/or analysed during the current study are available from the corresponding author on reasonable request.
