# Supplementary material for: Targeted high mean arterial pressure aggravates cerebral hemodynamics after extracorporeal resuscitation in swine
Source: Crit Care. 2021 Nov 14;25:369. doi: 10.1186/s13054-021-03783-3 (PMC8590749; doi:10.1186/s13054-021-03783-3)
Supplement: Supplementary file 1 — Additional file 1. Supplemental Table I: P values of the contingency table of the two-way analysis of variance (ANOVA) for repeated measures with group, time effect and group x time interaction effects for the different investigated parameters after cardiac arrest. [file 13054_2021_3783_MOESM1_ESM.docx]

**Supplemental Table I: P values of the contingency table of the two-way analysis of variance (ANOVA) for repeated measures with group, time effect and group x time interaction effects for the different investigated parameters after cardiac arrest.**

| Parameters | *P value* | | |
| --- | --- | --- | --- |
|  | **Group Effect** | **Time Effect** | **Group-Time Interaction** |
| Systemic hemodynamic |  |  |  |
| Heart rate (bpm) | 0.084 | 0.111 | 0.793 |
| Mean arterial pressure (mmHg) | 0.002 | 0.069 | 0.975 |
| Dose of adrenaline (µg/kg/min) | 0.071 | 0.108 | 0.371 |
| Cerebral hemodynamic |  |  |  |
| Intracranial pressure (cmH_2_O) | 0.134 | <0.0001 | 0.033 |
| Cerebral perfusion pressure (mmHg) | 0.137 | 0.010 | 0.839 |
| Cerebral blood flow (ml/min) | 0.117 | 0.034 | 0.006 |
| Pressure reactivity index | 0.683 | 0.193 | 0.018 |
| Cerebral oxygen consumption  (ml O_2_/min/kg, brain hemisphere) | 0.110 | 0.041 | 0.007 |
| NIRS cerebral oxygen saturation (%) | 0.329 | 0.025 | 0.145 |
| Metabolic |  |  |  |
| pH | 0.356 | <0.0001 | 0.001 |
| PaO_2_ (mmHg) | 0.385 | 0.529 | 0.076 |
| PaCO_2_ (mmHg) | 0.967 | <0.0001 | 0.422 |
| HCO_3_^-^ (mmol/L) | 0.685 | <0.0001 | 0.626 |
| Lactate (mmol/L) | 0.431 | 0.550 | 0.407 |

*MAP, mean arterial pressure; NIRS, near infrared spectroscopy; PaO_2,_ arterial oxygen partial pressure; PaCO_2_, arterial carbon dioxide partial pressure; IL-1β, interleukin-1β.*
